# Orexin Receptor Competitive Antagonists: A Novel target of the Sedative and hypnotics drugs for the pharmacotherapy of Insomnia

**DOI:** 10.3126/nje.v8i1.21139

**Published:** 2018-03-31

**Authors:** Indrajit Banerjee

**Affiliations:** 1 Associate Professor, Department of Pharmacology, SSR Medical College, Belle Rive, Mauritius.

Orexins are peptide neurotransmitters which are produced in the lateral and posterior part of the hypothalamus in the brain. There are two Orexin receptors which has been identified till date viz. Orexin 1 (OX 1) and Orexin 2 (OX 2 receptor). Orexins are associated with the sleep-wakefulness and it has been found in the experimental laboratory animals that deficient of Oxerin or the receptors for Orexin are found be associated with narcolepsy and disturbed sleep- awake pattern, day time sleepiness and cataplexy [1]. Orexins are also allied with feeding behaviors, autonomic function and reward in experimental laboratory animals[1]. Recently a number of drugs are targeted towards the Orexin receptors which is a recent target for the Sedative and hypnotics drugs for the treatment of Insomnia viz. Suvorexant, Almorexant, Lemborexant and Filorexant.

## Suvorexant

On 13 August 2014, Suvorexant has been approved by the US FDA as a new sedative and hypnotic drug acting as a competitive antagonist for the Orexin receptors and is found to be useful for the treatment of Insomnia. It was developed by Merck, and is marketed with the proprietary name Belsomra[2]. 

Mechanism of action: Suvorexant is found to be competitive antagonist towards the OX1 and OX 2 receptors. It is found to enhance the REM and NREM sleep in the insomniacs [3].

### Pharmacokinetic property

Suvorexant is well absorbed orally, takes approximately 2.2hours to reach the peak plasma concentration with a plasma half-life of twelve hours and the volume of distribution of about 105.9 L. It is having a high plasma protein binding capacity of 99.5%. There is no interaction of this drug with food intake. It undergoes metabolism by the Cytochrome P450 enzyme, mainly by CYP3A4 system and excreted through the feces.

Adverse drug effects: The reported adverse effects are day time somnolence, worsening of depression and suicidal ideation among individual[3].

Indication: Suvorexant is indicated in the pharmacotherapy of insomnia in adults of 18 years of age and older. Dose: The recommended daily dose is 10 mg, should be taken 30 mins before going to the bed.

### Clinical Trial Data

In a randomized controlled trial (RCT) conducted by Herring WJ et al. exhibited that patients received Suvorexant (62 patients received 10 mg, 61 patients 20 mg, 59 patients 40 mg or 61 patients 80 mg in one period and placebo for 249 patients for a period of 4 weeks increases sleep over the period of 4 weeks in patients with primary insomnia [4]. 

Report from another Randomized Controlled trial elucidated that Suvorexant promoted sleep but at higher therapeutic doses it can cause some residual effects [5].

## Almorexant

Mechanism of action: Almorexant is found to be a competitive antagonist towards the two Orexin receptors, Orexin1 and Orexin 2 receptor [6].

Data from a randomized controlled trial conducted by Black J et al revealed that it reduces the sleep latency and don’t cause any effect on hangover, rebound insomnia or withdrawal symptoms and dependence [7].

Dose: 200mg

Adverse effects were found to be similar to that of placebo.

## Lemborexant

Is found to be acting by blocking OX1 and 0X 2 receptors competitively. At a dose of 2.5-10 mg provided efficacy for the treatment of insomnia while minimizing the next-morning hangover.

In June 2016, Lemborexant is undergoing phase 3 clinical trials the USA, Germany, France, Italy, Poland, Spain, United Kingdom and Japan [8].

## Filorexant

In the year 2014, Filorexant has completed the phase II clinical trials. Filorexant is also an Orexin 1 and Orexin 2 receptor competitive antagonist. At the dose of 2.5/5/10/20mg is found to be more effective as compared to the placebo[9].

In conclusion Benzodiazepines and non benzodiazepines are prescribed commonly as a Sedative and hypnotic drugs for the treatment of insomnia. The adverse drug reactions and long term disadvantages has influenced the doctors and scientists to search for better options for the treatment of insomnia. The detection of orexins and their receptors has led to a colossal milestone in the field of Sedative and hypnotics drugs for the treatment of Insomnia. Suvorexant was approved by the US FDA as a new sedative and hypnotic drugs acting as a competitive antagonist of Orexin receptors and are found to be useful for the treatment of Insomnia. This has led to extensive research and numerous Randomized controlled trial to discover new drugs and compounds in this direction across the globe and undoubtedly these drugs will be the future drugs for the treatment of Insomnia.
